# Unpaid caregiving for people following hip fracture: longitudinal analysis from the English Longitudinal Study of Ageing

**DOI:** 10.1007/s41999-023-00843-5

**Published:** 2023-08-03

**Authors:** Toby O. Smith, S. Langford, K. Ward, R. Gray

**Affiliations:** 1 Warwick Medical School, University of Warwick, Coventry, CV4 7AL UK; 2 Faculty of Medicine and Health Sciences, University of East Anglia, Norwich, UK; 3 Department of Trauma and Orthopaedics, Northumbria Healthcare NHS Foundation Trust, Newcastle upon Tyne, UK; 4 Scottish Hip Fracture Audit, Public Health Scotland, Edinburgh, UK; 5 James Paget Hospital, Gorleston, Norfolk, UK

**Keywords:** Hip, Fracture, Care, Family, Recovery, Cohort analysis, National

## Abstract

**Purpose:**

To determine the provision and its change over time in unpaid care for people following hip fracture.

**Methods:**

Data were sought from the English Longitudinal Study of Ageing (ELSA) cohort. We identified participants who self-reported experiencing a hip fracture, who had clinical and caregiving data in the previous and subsequent two data collection waves. Demographic and clinical data were collected in addition to data on provision of unpaid care, who provided care and the frequency of needs being met.

**Results:**

The analysed cohort consisted of 246 participants [150 females (61%), mean age 78.9 years (standard deviation: 8.6)]. There was an increase in the number of participants requiring unpaid care between the Pre-Fracture and Fracture Wave (29% vs. 59%), which plateaued in the subsequent two waves (56%; 51%). Although both spouse and daughters provided the most unpaid care to participants over this study period, there was an increase in support provided during the Fracture Wave by both sons and daughters. This increased support offered by spouses continued until Post-Fracture Wave 2 when this plateaued. Support provided by friends increased from 3 to 8% and brothers and sisters increased from 0 and 1% Pre-Fracture to 8% by Post-Fracture Wave 2.

**Conclusion:**

These findings provide insights into who, what and how unpaid carers support people following hip fracture over time. Given the level of support unpaid carers offer, and previously reported carer stress and burden, undertaking clinical trials to assess the effectiveness of carer–patient support interventions would be valuable.

## Key summary points

**Aim:**

To determine the provision and its change over time in unpaid care for people following hip fracture.

**Findings:**

Unpaid care for people after hip fracture changes over time with an increase in the duration and network of providers offering this support. There is a shift towards providing greater personal care over time.

**Message:**

Health professionals should consider this wider network when supporting discharge planning of older people following hip fracture.

## Introduction

With a worldwide ageing population, the prevalence of hip fracture is increasing. By 2050, it is predicted that annually 4.5 million people will experience a hip fracture globally [1]. Whilst the number of people experiencing hip fracture is expected to be greatest in North America and Europe [2, 3], there is an anticipated rapid increase in prevalence in Asia and South America [4]. Whilst there have been advances in the treatment of hip fracture, with recommended standards developed globally [5–7], most focus on improvements related to inpatient care and hip fracture still confers high mortality and morbidity [8]. There is an increased risk of mortality for males, older people, those with greater pre-fracture care needs and people with multiple comorbidities [9].

Following hip fracture, individual’s physical capabilities, functional independence and health-related quality of life deteriorate [10, 11]. Friends and family members of those who sustain a hip fracture frequently provide support and care during the recovery period. These people are often referred to as informal or unpaid carers. They are expected to support the transition from hospital to home, facilitating patient’s ongoing recovery [12]. Tasks which unpaid carers may assist with range from personal activities of daily living (PADLs) such as toileting, washing, dressing and eating, to Incremental ADLS (IADLs) such as managing money, shopping and household chores [12].

There is a wealth of evidence reporting the high burden unpaid carers for people following hip fracture experience [12–14]. Carer burden has been attributed to a number of factors including: poor communication with health professionals [15, 16] and uncertainty on how best to support care-recipients [17]. However, there has been limited information detailing what activities unpaid carers do to support people following hip fracture, who they are and whether what they do changes over time.

The impact of social deprivation on the availability of unpaid carers is poorly understood. Those in the most deprived socioeconomic groups who sustain a hip fracture are younger at presentation, up to 5.6 years in one study [18]. The occupational status of carers, particularly a spouse, may have a greater bearing on readiness to provide support to their care-recipient. In addition, an individual’s ‘years spent in good health’ decreases progressively for those in the least-to-most deprived decile [19].

Previous literature on trajectories of unpaid care for older people suggests a gradual increase in both paid and unpaid care [20, 21]. Whilst this has been based on populations with diseases such as dementia and other chronic conditions [22, 23], it remains unclear whether this translates to the hip fracture population where there is a specific traumatic event which may suddenly change the capabilities of the person who sustained such as injury [24]. Furthermore, given the unexpected nature of trauma, the readiness of unpaid carers to their new role and responsibility can be difficult [24, 25]. Whether they continue in this role, or seek more paid sources of care, is unclear.

Given the uncertainty on unpaid carers for this population, the objective of this analysis was to determine:

- Who are the unpaid carers who provide support to people following hip fracture and does this change over time?
- What activities do unpaid carers support people following hip fracture and does this change over time?
- How much support do unpaid carers provide people following hip fracture and does this change over time?

## Methods

### Design and ethical issues

We reported a retrospective analysis of a prospective cohort study using the Strengthening the Reporting of Observational Studies in Epidemiology (STROBE) guidelines [26].

Data were sought from the English Longitudinal Study of Ageing (ELSA) cohort. This nationally representative cohort study commenced in 2002 and is an ongoing investigation into the health, social and economic lives of a cohort of people aged 50 years and older in England [27]. Data are collected every 2 years [27]. The anonymised ELSA datasets are publicly available for academic purposes. It was downloaded from the UK Data Service (https://ukdataservice.ac.uk/).

The English Longitudinal Study of Ageing was approved by the London Multicentre Research Ethics Committee (MREC/01/2/91). Participants gave informed consent.

### Participants

We identified participants who self-reported an initial (first-time) hip fracture within a given data collection wave [ELSA Wave 2 (2004–2005) to Wave 7 (2014–2015)]. This permitted data to be gathered within a two wave (4-year) follow-up period following the hip fracture wave (termed ‘Fracture Wave’).

### Exposure variables

We gathered data from the period of the fracture (Fracture Wave) in addition to the preceding wave (Pre-Fracture Wave) and two subsequent waves (Post-Fracture Wave 1/Post-Fracture Wave 2).

To understand the characteristics of the analysed cohort, we gathered data at the Fracture Wave on age, sex, marital status, whether participants lived alone, ethnicity and socioeconomic status measured using the National Statistics Socio-economic Classification-5 item (NS-SeC5) [28].

To understand clinical status at all data collection waves, we gathered data on pain severity, difficulty walking 100 yards, frequency of common medical comorbidities (e.g. diabetes, stroke, hypertension, arthritis, depression), number of falls and whether a fall required medical attention, perceived self-reported health, and the Control, Autonomy, Self-Realization and Pleasure-19 (CASP-19) [29] total score to measure health-related quality of life (HRQoL).

### Outcome variables

To assess changing care needs, in all four assessment waves, we collected data on whether participants received unpaid or paid care, requirement for assistance with PADLs (e.g. dressing, washing, eating, and toileting) or IADLs (e.g. shopping, administering medicines, and managing money), number of activities requiring unpaid care, who provided unpaid care (e.g. spouse, child, grandchild, neighbour, and friend) and the frequency of needs being met. To determine whether a participant received or did not receive unpaid care, we attributed a composite of the response to: (1) the number of unpaid carers assisting and (2) requirement for assistance. We also collected data to determine whether a change of residential status occurred due to health (and if to institutional care) within a given wave.

### Statistical analysis

ELSA longitudinal weights are only available for core sample participants. Applying longitudinal weights to our analysis would have resulted in a reduction in analytical sample size and, therefore, reduced statistical power. We, therefore, used the unweighted sample for our analyses.

Descriptive statistics (mean, standard deviation (SD), frequencies and percentages) were used to profile the analysed cohort and present the change in care requirements over the assessment waves. We did not analyse data inferentially. This was justifiable given the number of missing participants in Post-Fracture Wave 1 and Wave 2 increased the risk of type two statistical error, given the number of variables analysed. Data were presented as line graphs across the four time-points to illustrate changes in unpaid care requirements and change in the composition of care networks over time.

Analyses were performed using Stata/MP 17.0 for Windows (StataCorp LLC, Texas 77845, USA).

## Results

### Cohort characteristics

**Fig. 1:**
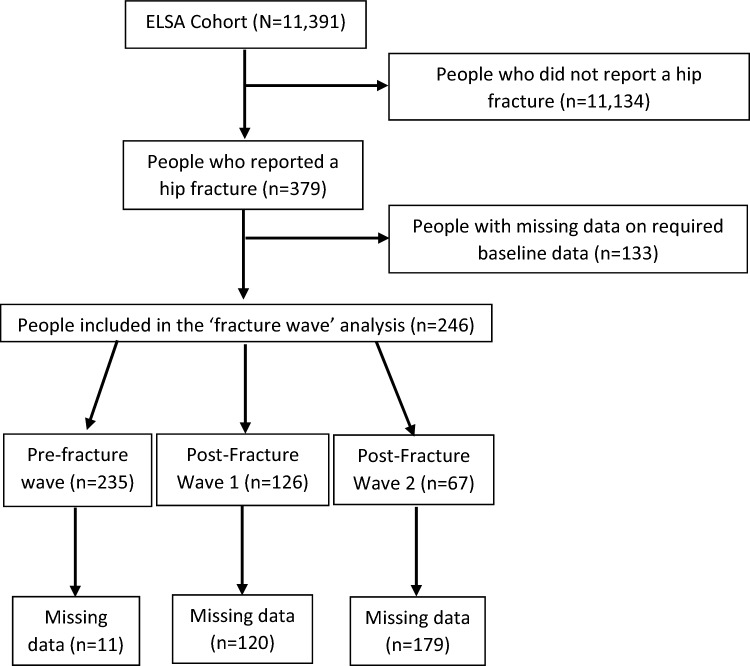
Flow chart illustrating cohort composition from respective datasets

Figure 1 illustrates where the study cohort derived from the overall ELSA dataset. In total, 379 participants who sustained a hip fracture were identified. Of these, 133 participants had missing baseline demographic characteristics or caregiving requirement data. This resulted in a Fracture Wave cohort of 246 participants. From this cohort, there were data on caregiving requirements for 235 in the Pre-Fracture Wave, 126 from Post-Fracture Wave 1 and 67 from Post-Fracture Wave 2.

The characteristics of the cohort at their Fracture Wave are presented in Table 1. This illustrates the cohort of 246 participants consisted of 150 females (61%) with a mean age of 78.9 (SD 8.6) years. Thirty-seven percent of the cohort were married and 32% lived alone. Fifty-one percent self-reported difficulties walking 100 yards; 35% reported moderate or severe pain. Pain severity was not reported in 52% of the cohort. At the Fracture Wave, 59% reported receiving unpaid care, whereas 23% reported receiving paid care either in isolation or in addition to unpaid care.

**Table 1:** Characteristics of the hip fracture cohort within the fracture wave

	Total analysed cohort
*N*	246
Gender	
Male	96 (39.0)
Female	150 (61.0)
Age (mean; SD)	78.9 (8.6)
Marital status	
Single	9 (3.7)
Married	90 (36.6)
Divorced	20 (8.1)
Remarried	24 (9.8)
Widowed	103 (41.9)
Lives alone (yes; %)	78 (31.7)
Ethnicity	
White	243 (98.8)
Asian	2 (0.8)
Black	1 (0.4)
Other	0 (0.0)
NS-SEC5	
Managerial and professional occupations	59 (24.0)
Intermediate	23 (9.3)
Low supervisory and technical	17 (6.9)
Semi-routine	62 (25.2)
Small employers and own account workers	12 (4.9)
Not in occupation	73 (29.7)
Difficulty walking 100 yards (yes; %)	125 (50.8)
Pain severity	
Mild	32 (13.0)
Moderate	54 (22.0)
Severe	31 (12.6)
Not reported	129 (52.4)
Care receipt	
Received unpaid care (yes; %)	145 (58.9)
Received paid care (yes; %)	56 (22.6)
Assistance with (yes; %)	
Dressing	67 (27.2)
Walking	31 (12.6)
Washing	73 (29.7)
Eating	28 (11.4)
Bed transfers	33 (13.4)
Toileting	23 (9.3)
Shopping	102 (41.5)
Medicines	42 (17.1)
Housework	120 (48.8)
Managing money	58 (23.6)
Assistance provided (yes; %)	
Spouse/partner	42 (17.1)
Son	32 (13.0)
Daughter	51 (20.7)
Grandchild	8 (3.3)
Sister	6 (2.4)
Brother	3 (1.2)
Other relative	7 (2.8)
Friend	17 (6.9)
Neighbour	10 (4.1)
Number activities seek assistance for (mean; SD)	2.8 (3.6)
Self-reported health	
Excellent	9 (3.7)
Very good	32 (13.0)
Fair	71 (28.9)
Poor	61 (24.8)
Very bad	0 (0.0)
Not stated	73 (29.6)
Reported morbidities (yes; %)	
Angina	8 (3.3)
Congestive cardiac failure	1 (0.4)
Diabetes	31 (12.6)
Stroke	13 (5.3)
Hypertension	107 (43.5)
Cancer	14 (5.7)
COPD	15 (6.1)
Asthma	28 (11.4)
Arthritis	111 (45.1)
Dementia	4 (1.6)
Parkinson’s	3 (1.2)
Anxiety	11 (4.5)
Depression	9 (3.7)
Falls	
Number falls (mean; SD)	1.6 (4.0)
Falls requiring hospitalisation (yes; %)	101 (41.1)
CASP-19 total score (mean; SD)	37.1 (6.2)
Residential status change	
Moved for health reasons (yes; %)	6 (2.4)
Moved into a nursing or residential home (yes; %)	1 (0.4)

Participants could have multiple activities requiring assistance or carers providing assistance, therefore, data reflect the frequency to-which these occurred within each cohort

*CASP* Control, Autonomy, Self-Realization and Pleasure-19 questionnaire, *COPD* chronic obstructive pulmonary disease, *N* number of participants, *SD* standard deviation

### Changes in clinical and social status post-hip fracture

Table 2 illustrates the change in clinical and social outcomes for the cohort prior to, during the Fracture Wave and in Post-Fracture Wave 1 and Wave 2. Over these latter waves, 12% of the cohort experienced a second hip fracture within Post-Fracture Wave 1, whilst 8% reported this event in Post-Fracture Wave 2. There was a trend for better health outcomes pre-fracture compared to the subsequent waves. The greatest reported disability, measured through difficulty walking (51% vs. 42%, vs. 48%), pain severity (severe pain: 13% vs. 14% vs. 12%) and reported ‘good’ or ‘very good’ self-reported health (43% vs 40% vs. 31%) occurred in the Fracture Wave compared to two subsequent waves. This did not substantially change over time. Similarly, there was limited difference in the presentation of comorbidities across the study period (Table 2). Mean CASP-19 score did not substantially change between the time-points (mean: 37.1 to 36.8 points). The number of people who moved into residential or nursing home care because of health reasons was small with limited change over time (Table 2).

**Table 2:** Trajectory of unpaid care needs (and characteristics) before and after hip fracture

	Pre-Fracture Wave	Fracture Wave	Post-Fracture Wave 1	Post-Fracture Wave 2
*N*	235	246	126	67
Hip fracture (yes; %)	0 (0.0)	246 (100.0)	15 (11.9)	5 (7.5)
Difficulty walking 100 yards (yes; %)	60 (25.5)	125 (50.8)	53 (42.1)	32 (47.8)
Pain severity (yes; %)				
Mild	22 (9.4)	32 (13.0)	15 (11.9)	9 (13.4)
Moderate	35(14.9)	54 (22.0)	35 (27.8)	21 (31.3)
Severe	23(9.8)	31 (12.6)	18 (14.3)	8 (11.9)
Not reported	155 (66.0)	129 (52.4)	58 (46.0)	29 (43.3)
Lives alone (yes; %)	72 (30.6)	78 (31.7)	41 (32.5)	25 (37.3)
Care receipt				
Received unpaid care (yes; %)	67 (28.5)	145 (58.9)	70 (55.5)	34 (50.7)
Received paid care (yes; %)	53 (22.7)	56 (22.6)	12 (9.5)	3 (4.6)
Assistance with (yes; %)				
Dressing	32 (13.6)	67 (27.2)	32 (25.4)	10 (14.9)
Walking	28 (11.9)	31 (12.6)	16 (12.7)	6 (9.0)
Washing	29 (12.3)	73 (29.7)	33 (26.2)	18 (26.9)
Eating	15 (6.4)	28 (11.4)	17 (13.5)	6 (9.0)
Bed transfers	7 (3.0)	33 (13.4)	19 (15.1)	7 (10.4)
Toileting	4 (1.7)	23 (9.3)	14 (11.1)	17 (25.4)
Shopping	76 (32.3)	102 (41.5)	54 (42.9)	24 (35.8)
Medicines	10 (4.3)	42 (17.1)	19 (15.1)	14 (20.9)
Housework	82 (34.9)	120 (48.8)	59 (46.8)	28 (41.8)
Managing money	30 (12.8)	58 (23.6)	22 (17.5)	13 (19.4)
Number activities need assistance (mean; SD)	1.7 (2.4)	2.8 (3.6)	2.7 (3.6)	2.1 (3.0)
Assistance provided by (yes; %)				
Spouse/partner	27 (11.5)	42 (17.1)	23 (18.3)	11 (16.4)
Son	16 (6.8)	32 (13.0)	12 (9.5)	2 (3.0)
Daughter	30 (12.8)	51 (20.7)	24 (19.0)	11 (16.4)
Grandchild	5 (2.1)	8 (3.3)	8 (6.3)	4 (6.0)
Sister	2 (0.9)	6 (2.4)	4 (3.2)	5 (7.5)
Brother	0 (0.0)	3 (1.2)	0 (0.0)	5 (7.5)
Other relative	5 (2.1)	7 (2.8)	3 (2.4)	1 (1.5)
Friend	8 (3.4)	17 (6.9)	10 (7.9)	5 (7.5)
Neighbour	2 (0.9)	10 (4.1)	1 (0.8)	0 (0.0)
Self-reported health (yes; %)				
Excellent	8 (3.4)	9 (3.7)	6 (4.8)	5 (7.5)
Very good	28 (11.9)	32 (13.0)	17 (13.5)	6 (9.0)
Good	49 (20.9)	71 (28.9)	33 (26.2)	15 (22.4)
Fair	56 (23.8)	61 (24.8)	38 (30.2)	18 (26.9)
Poor	21 (8.9)	0 (0.0)	18 (14.3)	7 (10.4)
Very bad	0 (0.0)	33 (13.4)	0 (0.0)	8 (11.9)
Not stated	73 (31.1)	40 (16.3)	14 (11.1)	8 (11.9)
Needs met (yes; %)				
Sometimes	7 (3.0)	7 (2.8)	4 (3.2)	23 (34.3)
Usually	14 (6.0)	24 (9.8)	16 (12.7)	7 (10.4)
All time	57 (24.3)	110 (44.7)	47 (37.3)	37 (55.2)
Not reported	157 (66.8)	105 (42.7)	59 (46.8)	0 (0.0)
Comorbidities reported (yes; %)				
Angina	9 (3.8)	8 (3.3)	1 (0.8)	4 (6.0)
Congestive cardiac failure	0 (0.0)	1 (0.4)	0 (0.0)	0 (0.0)
Diabetes	24 (10.2)	31 (12.6)	19 (15.1)	9 (13.4)
Stroke	12 (5.1)	13 (5.3)	8 (6.3)	5 (7.5)
Hypertension	86 (36.6)	107 (43.5)	48 (38.1)	24 (35.8)
Cancer	7 (3.0)	14 (5.7)	5 (4.0)	4 (6.0)
COPD	9 (3.8)	15 (6.1)	4 (3.2)	2 (3.0)
Asthma	19 (8.1)	28 (11.4)	16 (12.7)	6 (9.0)
Arthritis	81 (34.5)	111 (45.1)	59 (46.8)	22 (32.8)
Dementia	2 (0.9)	4 (1.6)	0 (0.0)	1 (1.5)
Parkinson’s	2 (0.9)	3 (1.2)	3 (2.4)	2 (3.0)
Anxiety	6 (2.6)	11 (4.5)	2 (1.6)	1 (1.5)
Depression	7 (3.0)	9 (3.7)	5 (4.0)	1 (1.5)
Falls				
Number falls (mean; SD)	2.4 (9.2)	1.6 (4.0)	1.2 (2.6)	1.0 (1.8)
Falls requiring hospitalisation (yes; %)	38 (16.2)	101 (41.1)	17 (13.5)	13 (19.4)
CASP-19 Total Score (mean; SD)	36.2 (5.8)	37.1 (6.2)	36.8 (6.3)	36.9 (4.5)
Residential status change				
Moved home because of health reasons (yes; %)	3 (1.3)	6 (2.4)	2 (1.6)	1 (1.5)
Moved into a nursing or residential home (yes; %)	2 (0.9)	1 (0.4)	1 (0.8)	1 (1.5)

Participants could have multiple activities requiring assistance or carers providing assistance, therefore, data reflect the frequency to-which these occurred within each cohort

*COPD* chronic obstructive pulmonary disease, *N* number of participants, *SD* standard deviation

### Changes in caregiving requirements and delivery post-hip fracture

**Fig. 2:**
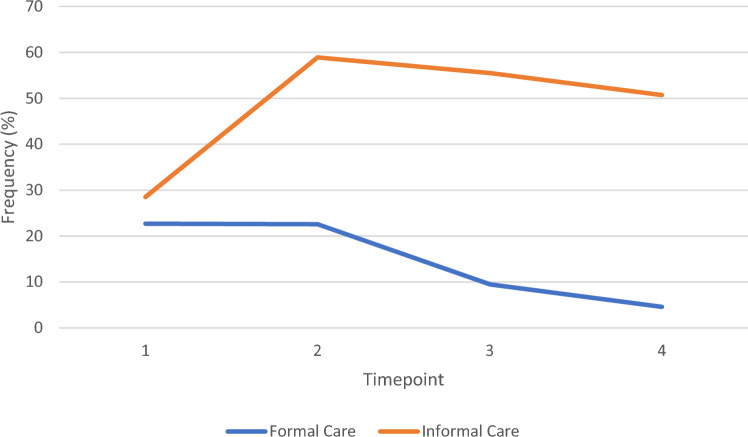
Line graph to illustrate change in paid and unpaid care received from the Pre-Fracture Wave to second Post-Fracture Wave

There was an increase in the number of participants requiring unpaid care between the Pre-Fracture and Fracture Wave (29% vs. 59%). This remained consistent in the subsequent two waves (56% and 51%; Fig. 2). As illustrated in Fig. 2, the proportion of people receiving paid care decreased from the Fracture Wave (23%) to the Post-Fracture Wave 1 (10%) and Post-Fracture Wave 2 (5%).

**Fig. 3:**
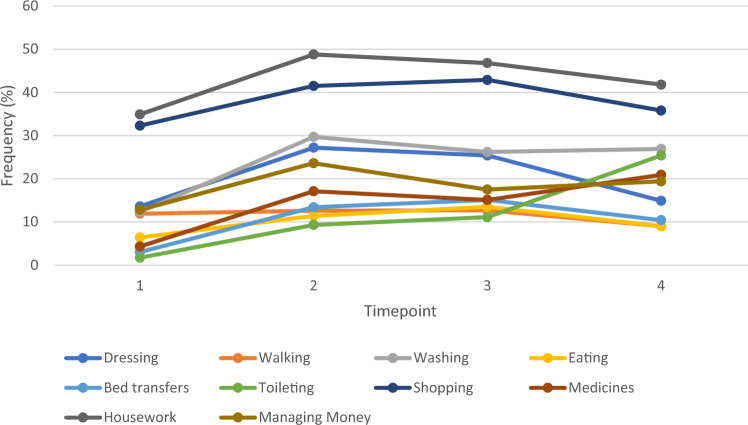
Line graph to illustrate the change in unpaid care by care need received from pre-fracture wave to second Post-Fracture Wave

There was an overall trend in requiring increased unpaid care for both PADLs and IADLs from the Pre-Fracture to Fracture Wave. As Fig. 3 illustrates, whilst participants reported an increase in unpaid care for all activities except ‘walking’, these decreased over subsequent waves except for assistance with toileting (9% vs. 11% vs. 25%). There was an increase in requiring assistance for administering medicines (15% to 21%) from Post-Fracture Wave 1 to Wave 2 (Fig. 3).

**Fig. 4:**
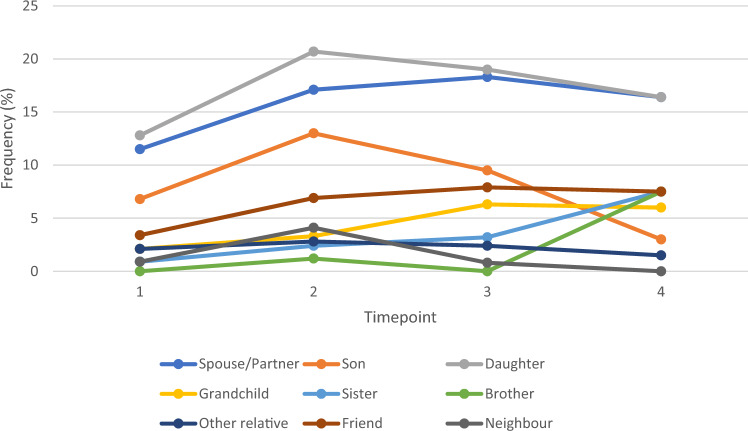
Line graph to illustrate the change in provider of unpaid care received from Pre-Fracture Wave to second Post-Fracture Wave

As Fig. 4 illustrates, although both spouse and daughters provided the most unpaid care over this study period, the support they offered differed over time. Whilst there was an increase in support provided during the Fracture Wave by both sons and daughters, the increased support offered by spouses continued until Post-Fracture Wave 2 when this plateaued. Overall, there was an increase in support offered by wider friends and family members over time. Support provided by friends increased from 3 to 8% and support from brothers and sisters increased from 0 and 1% Pre-Fracture to 8% for both by Post-Fracture Wave 2.

**Fig. 5:**
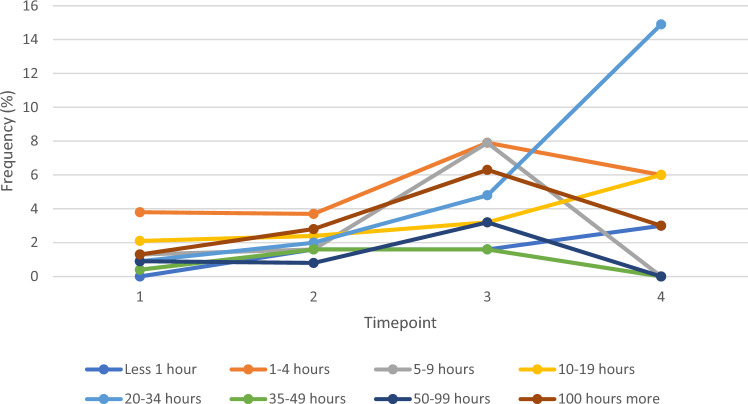
Line graph illustrating the change in duration of unpaid caregiving performed by spouse/partner from Pre-Fracture Wave to the second Post-Fracture Wave

Figure 5 illustrates the duration of unpaid care provided by spouses/partners over the study period. As this illustrates, whilst there was an increase from the Fracture Wave to Post-Fracture Wave 1 for most categorises of duration, these largely decreased from Post-Fracture Wave 1 to Post-Fracture Wave 2 (Table 3). The exception to this was for lower duration periods, namely less than 1 h (2 to 3%), 10 to 19 h (2 to 6%) and 20 to 34 h (2 to 15%) between Fracture Wave and Post-Fracture Wave 2.

**Table 3:** Hours of caregiving across waves

	Less than 1 h	1–4 h	5–9 h	10–19 h	20–34 h	35–49 h	50–99 h	100 h or more
Pre-fracture wave (*n* = 235)								
Partner/spouse	0 (0.0)	9 (3.8)	3 (1.3)	5 (2.1)	2 (0.9)	1 (0.4)	2 (0.9)	3 (1.3)
Daughter	0 (0.0)	8 (3.4)	3 (1.3)	0 (0.0)	0 (0.0)	0 (0.0)	0 (0.0)	0 (0.0)
Son	0 (0.0)	14 (6.0)	5 (2.1)	2 (0.9)	4 (1.7)	0 (0.0)	2 (0.9)	1 (0.4)
Grandchild	0 (0.0)	6 (2.6)	0 (0.0)	0 (0.0)	0 (0.0)	0 (0.0)	0 (0.0)	0 (0.0)
Sister	0 (0.0)	1 (.04)	0 (0.0)	0 (0.0)	0 (0.0)	0 (0.0)	0 (0.0)	0 (0.0)
Brother	0 (0.0)	1 (0.4)	0 (0.0)	0 (0.0)	0 (0.0)	0 (0.0)	0 (0.0)	0 (0.0)
Other relative	0 (0.0)	4 (1.7)	0 (0.0)	1 (0.4)	0 (0.0)	0 (0.0)	0 (0.0)	0 (0.0)
Friend	0 (0.0)	5 (2.1)	1 (0.4)	1 (0.4)	0 (0.0)	0 (0.0)	0 (0.0)	0 (0.0)
Neighbour	0 (0.0)	1 (0.4)	1 (0.4)	0 (0.0)	0 (0.0)	0 (0.0)	0 (0.0)	0 (0.0)
Fracture wave (*n* = 246)								
Partner/spouse	4 (1.6)	9 (3.7)	4 (1.6)	6 (2.4)	5 (2.0)	4 (1.6)	2 (0.8)	7 (2.8)
Daughter	11 (4.5)	9 (3.7)	2 (0.8)	2 (0.8)	0 (0.0)	0 (0.0)	0 (0.0)	0 (0.0)
Son	1 (0.4)	15 (6.1)	10 (4.1)	11 (4.5)	11 (4.5)	2 (0.8)	3 (1.2)	3 (1.2)
Grandchild	1 (0.4)	2 (0.8)	3 (1.2)	3 (1.2)	1 (0.4)	0 (0.0)	0 (0.0)	0(0.0)
Sister	4 (1.6)	1 (0.4)	0 (0.0)	0 (0.0)	0 (0.0)	0 (0.0)	0 (0.0)	0 (0.0)
Brother	1 (0.4)	0 (0.0)	0 (0.0)	0 (0.0)	0 (0.0)	0 (0.0)	0 (0.0)	0 (0.0)
Other relative	2 (0.8)	3 (1.2)	1 (0.4)	0 (0.0)	0 (0.0)	0 (0.0)	0 (0.0)	0 (0.0)
Friend	2 (0.8)	25 (10.2)	8 (3.3)	0 (0.0)	2 (0.8)	0 (0.0)	0 (0.0)	0 (0.0)
Neighbour	1 (0.4)	9 (3.7)	2 (0.8)	1 (0.4)	0 (0.0)	0 (0.0)	0 (0.0)	0 (0.0)
Post-Fracture Wave 1 (*n* = 126)								
Partner/spouse	2 (1.6)	10 (7.9)	10 (7.9)	4 (3.2)	6 (4.8)	2 (1.6)	4 (3.2)	8 (6.3)
Daughter	0 (0.0)	7 (5.6)	2 (1.6)	0 (0.0)	0 (0.0)	0 (0.0)	2 (1.6)	0 (0.0)
Son	2 (1.6)	12 (9.5)	5 (4.0)	4 (3.2)	1 (0.8)	0 (0.0)	1 (0.8)	0 (0.0)
Grandchild	0 (0.0)	2 (1.6)	3 (2.4)	1 (0.8)	1 (0.8)	0 (0.0)	0 (0.0)	0 (0.0)
Sister	0 (0.0)	3 (2.4)	1 (0.8)	0 (0.0)	0(0.0)	0 (0.0)	0 (0.0)	0 (0.0)
Brother	0 (0.0)	0 (0.0)	0 (0.0)	0 (0.0)	0 (0.0)	0 (0.0)	0 (0.0)	0 (0.0)
Other relative	0 (0.0)	3 (2.4)	1 (0.8)	0 (0.0)	0 (0.0)	0 (0.0)	0 (0.0)	0 (0.0)
Friend	1 (0.8)	8 (6.3)	1 (0.8)	0 (0.0)	2 (1.6)	0 (0.0)	0 (0.0)	0 (0.0)
Neighbour	0 (0.0)	0 (0.0)	0 (0.0)	0 (0.0)	0 (0.0)	0 (0.0)	0 (0.0)	0 (0.0)
Post-Fracture Wave 2 (*n* = 67)								
Partner/spouse	2(3.0)	4 (6.0)	0 (0.0)	4 (6.0)	10 (14.9)	0 (0.0)	0 (0.0)	2 (3.0)
Daughter	0 (0.0)	2 (3.0)	0 (0.0)	0 (0.0)	0 (0.0)	0 (0.0)	0 (0.0)	0 (0.0)
Son	0 (0.0)	7 (10.4)	3 (4.5)	1 (1.5)	0 (0.0)	1 (1.5)	0 (0.0)	1(1.5)
Grandchild	0 (0.0)	1 (1.5)	0 (0.0)	1 (1.5)	2 (3.0)	0 (0.0)	0 (0.0)	0 (0.0)
Sister	0 (0.0)	0 (0.0)	0 (0.0)	0 (0.0)	0 (0.0)	0 (0.0)	0 (0.0)	0 (0.0)
Brother	0 (0.0)	0 (0.0)	0 (0.0)	0 (0.0)	0 (0.0)	0 (0.0)	0 (0.0)	0 (0.0)
Other relative	0 (0.0)	0 (0.0)	0 (0.0)	0 (0.0)	0 (0.0)	0 (0.0)	0 (0.0)	0 (0.0)
Friend	3 (4.5)	3 (4.5)	0 (0.0)	0 (0.0)	0 (0.0)	0 (0.0)	0 (0.0)	0 (0.0)
Neighbour	0 (0.0)	0 (0.0)	0 (0.0)	0 (0.0)	0 (0.0)	0 (0.0)	0 (0.0)	0 (0.0)

*Data presented as frequency and percentages

## Discussion

These findings indicate that whilst unpaid care needs increased around the time of an individual’s hip fracture, this plateaued or steadily decreased over the 4 years following fracture. Whilst unpaid care was largely delivered by a spouse or the person’s child at the time of fracture, over time, there was increased delivery of unpaid care from a wider caregiving network including siblings and friends. In the period around a hip fracture, these people frequently received unpaid care for PADLs such as washing, dressing and eating/food preparation. Whilst this decreased over time, there was increased support received for more complex tasks such as managing money, administering medications and more physically demanding activities such as housework. Although the frequency of unpaid care plateaued after hip fracture, there were indicative findings that the duration of care a spouse offered changed over time, with reduced durations over time, potentially because of a wider caregiving network becoming engaged.

The results suggest an improvement in pain and reduced disability 2 and 4 years post-fracture. This conflicts with previous literature suggesting clinical status following hip fracture deteriorates post-fracture [30, 31]. These findings may be attributed to attrition within the cohort. There may be differential loss to follow-up for those with the worse outcomes. Consequentially, the Post-Fracture Wave 1 and Wave 2 cohorts may have an inflated health status for this population. Given the mean age of the cohort at the Fracture Wave was 79 years, this would be a reasonable assumption. Based on this, the interpretation of carer’s needs may be viewed with caution. Further exploration based on an inception cohort, specifically designed to assess caregiving following hip fracture, would be warranted to explore whether additional variables such as associated morbidity and mortality, disability, access to care and other covariates were influenced by attrition.

Unpaid care activities increased after hip fracture. This mirrors previous understanding where people require help and support during their early recovery after a hip fracture [24, 32]. Hip fracture, therefore, may be seen as a ‘trigger event’, to instigate the offer of training and support to those who were not previously unpaid carers or those who need added guidance. The literature has repeatedly demonstrated that these ‘new’ unpaid carers can struggle with the role and responsibility that they may be suddenly placed under [24]. These findings reinforce the notion that the occurrence of a hip fracture should ‘flag’ to health professionals a global assessment of patient’s and unpaid carer’s needs, to precipitate the provision of appropriate support. Such interventions may reduce potential carer anxiety, stress and burden [12, 14]. Whilst clinical trials such as HIP HELPER [17] are beginning to explore this, further research to understand effective means of identifying unpaid carers, determining their needs and delivering education and training are required to ensure that both they and the person with a hip fracture, are supported, and particularly if different methods are required depending on age, time-commitments outside of caregiving and technological aptitude of the unpaid carer. This would provide further empirical evidence to the recommendations made by de Lima et al. [33] who supported the need to formal educational courses to empower carers to more effectively support those they care for, whilst reducing overarching burden and anxiety which can be experienced by unpaid carers [12–14].

As reported previously, hip fracture is unfortunately not always a ‘one-off’ event [34]. In this cohort, 12% experienced a second hip fracture within 2 years of the first; a further 8% from 2 to 4 years. These people may, therefore, have continuing care needs long after the initial hip fracture. This is illustrated by our data that whilst unpaid care overall, and for the specific activities reported, decreased from the Fracture Wave to subsequent waves, unpaid care was provided more frequently than pre-fracture. To maintain this support, whilst spouse/partner and children are the principal providers of unpaid care, a wider network may develop over time from grandchildren and friends. This is also reported in other populations such as unpaid carers of people with dementia [22] and other chronic diseases [23]. Acknowledgement of this wider network is important. Health professionals should be mindful that focussing on close relatives may not necessarily be the optimal approach in providing unpaid carer support. Assumptions in perceived gender-typical roles of carers should be avoided. Individually assessing a care-recipient’s network, and tailoring guidance and support, to ensure all members of the care network are supported, should be considered.

Whilst this is the first study to offer insights on changing unpaid carer’s needs for people following hip fracture, further research is now warranted to provide more granular detail of unpaid care for this population. The ELSA study offers important care data, but the two-yearly waves meant data on what happens in-between these waves were unavailable. Furthermore, there were less data on carers themselves and the impact of caregiving on them. Further exploration of these important questions is now warranted given the current findings indicate change occurs over time for these people, and caregiving activity are common in this population.

Whilst this study provides important data from a nationally representative cohort on unpaid care following hip fracture, there are three important limitations which should be considered when interpreting these findings. First, it was not possible to determine the actual date of hip fracture. The ELSA data are presented in two-yearly ‘waves’. A hip fracture may, therefore, have occurred at any point within that wave. This may have modified the disability, and subsequent care requirements reported by participants. Analysing by the actual date of fracture was not possible as this was not provided. Nonetheless, the data provide valuable caregiving insights over time, which had not been previously reported. Second, because of participant loss to follow-up, there were a smaller number of participants in each Post-Fracture Wave. Consequentially, analysis through inferential tests was inappropriate and underpowered. Whilst the descriptive statistical analysis offered important insights, the adoption of statistical analyses may provide greater certainty on the interpretations. Exploration of factors associated with these changes over time, using multi-level modelling, would be advisable with a larger cohort. Finally, the cohort predominantly self-identified as ‘white’ and, therefore, may not be representative of some communities in the UK, let alone globally. Previous literature suggests the role of unpaid care and supporting family members and friends following health events differs based on religious, cultural and social beliefs [35, 36]. Further exploration of different communities and populations would advance knowledge on unpaid care following hip fracture.

To conclude, people following hip fracture receive increasing levels of unpaid care following their trauma, to support their ability to undertake ADLs at home compared to pre-fracture requirements. Whilst spouse and close family members offer support, the care network increases with time across wider family members and friends. Understanding this important network and providing support to these people, may improve health and well-being outcomes for those following hip fracture and their unpaid carers. Given the level of support unpaid carers offer, and previously reported carer stress and burden, undertaking clinical trials to assess the effectiveness of carer–patient support interventions would be valuable.

## Data Availability

The data that support the findings of this study may be available from the corresponding author (TOS) upon reasonable request.

## Declarations

### Submission declaration and verification

The authors declare that this work has not been previously published, that it is not under consideration for publication elsewhere, and that its publication has been approved by all the authors and tacitly or explicitly by the responsible authorities where the work was carried out and that, if accepted, it is not it will be published elsewhere in the same form, in English or in any other language, including electronically, without the written consent of the copyright holder.
